# Germ Cell Tumor of the Yolk Sac in the Uterine Corpus: Case Report of a 14-Month-Old Female Infant

**DOI:** 10.7759/cureus.50737

**Published:** 2023-12-18

**Authors:** Amaranto Suárez, Javier Brito Moreno, Maria Camila Suaza Vallejo, Juan Pablo Luengas, Carlos Blanco

**Affiliations:** 1 Pediatric Oncology, Instituto Nacional de Cancerología, Bogotá, COL; 2 Pediatric Hematology and Oncology, Instituto Nacional de Cancerología, Bogotá, COL

**Keywords:** germ cell, neoplams, prepuberty, uterus, extragonadal, yolk sac tumor

## Abstract

Malignant germ cell tumors (MGCTs) localized in the uterus are rare in prepubertal girls. They typically occur in postmenopausal women and are characterized by the presence of a pelvic mass and transvaginal bleeding. In this case, the authors describe the clinical features, radiologic findings, histopathologic description, and treatment received by an infant with a primary yolk sac tumor of the uterine wall. Currently, treatment for uterine GCTs is based on guidelines for GCTs. Surgery and bleomycin, etoposide, cisplatin (pBEP) chemotherapy are effective for uterine yolk sac tumors. After 46 months of clinical follow-up, which included abdominopelvic ultrasound and tumor marker assessments, our patient is free of disease, suggesting a favorable outcome.

## Introduction

Malignant germ cell tumors (MGCTs) account for 2% to 5% of all cancers in children under 15 years of age and represent around 14% of tumors in adolescents between 15 and 19 years of age [1]. According to Surveillance, Epidemiology, and End Results (SEER) data, in the United States between 2015 and 2019, in registries covering 66% of all children in the United States, there was an incidence of extracranial and extragonadal GCTs of 1.39 per million females under 20 years of age and 1.25 per million in males [2]. Although MGCTs frequently originate in the gonads, they can also develop in extragonadal sites such as the midline, sacrococcygeal region, mediastinum, retroperitoneum, and female reproductive tract [3]. The occurrence of a GCT in the uterus is very rare in the pediatric age group. Yin M et al., in 2022, published a case of a two-year-old girl with a uterine yolk sac tumor and reported, as of the date of publication, 27 cases in women aged between 27 and 87 years [4].

## Case presentation

**Figure 1:**
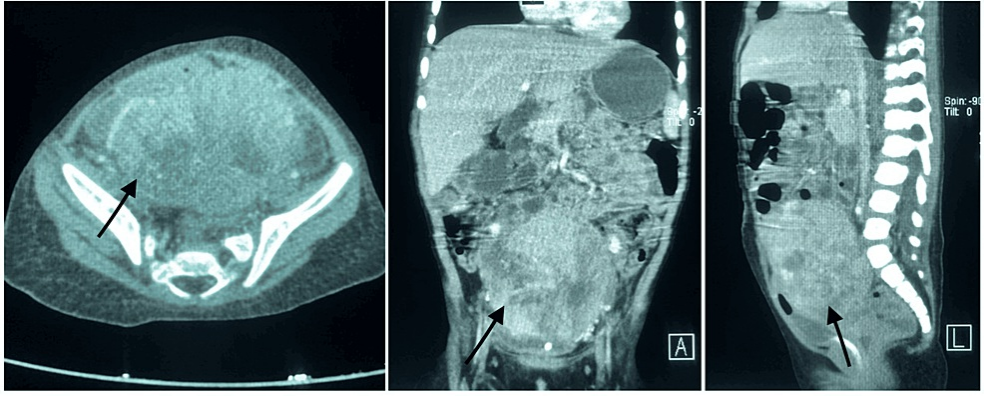
CT images at diagnosis. Dual-contrast CT scan of the abdomen and pelvis shows a heterogeneous mass with punctate calcifications, measuring 68x70x60 mm, centered in the uterus. The mass surrounds the left external iliac artery approximately 180° and the internal iliac artery 90°, without any cleavage planes. The scan also reveals a displaced and collapsed bladder. No lesions suggestive of liver metastasis were observed (see arrows).

We report a case of a 14-month-old girl without a medical record who visited the hospital for several weeks of transvaginal bleeding associated with an increase in abdominal volume. The abdominal ultrasound evidenced a solid mass at the pelvic level and left hydronephrosis, grade II. The abdominopelvic CT scan showed an enlarged uterus deformed by a mass measuring 68 x 70 x 60 mm, with no cleavage plane that occupied the abdominal cavity (Figure 1). At the time of diagnosis, tumor markers were elevated, with α-fetoprotein over 50,000 ng/ml and β-human chorionic gonadotropin at 0.32 IU/ml. The tumor was considered unresectable at the time of diagnosis, so vaginoscopy and tumor biopsy were performed.

**Figure 2:**
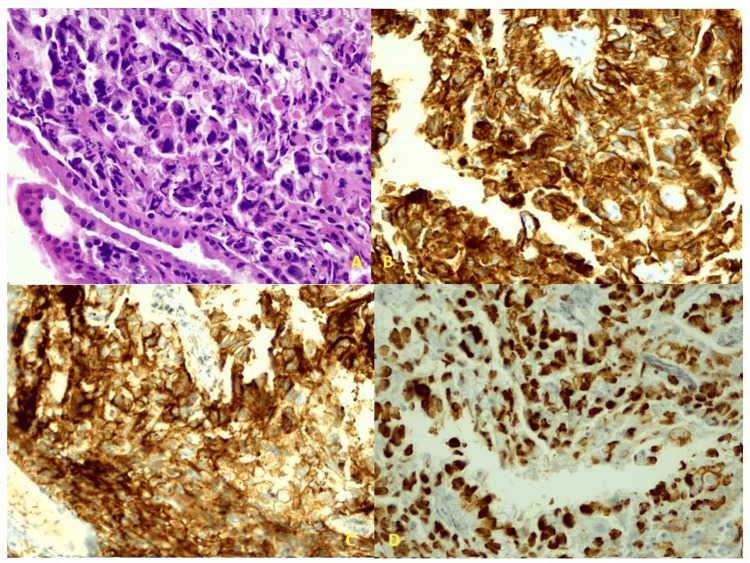
Histopathology and immunohistochemistry image of biopsy specimen. A. Pathology reports a malignancy consisting of a solid mass of large, atypical cells with abundant clear cytoplasm and areas of glandular proliferation lined by columnar cells or squamous epithelia. B. Positive immunohistochemistry for AE1/AE3. C. Positive immunohistochemistry for α-fetoprotein. D. SALL4 and glypican-3 were also positive on immunohistochemistry.

The pathology report revealed a malignant tumor characterized by a solid proliferation of large, atypical cells with abundant clear cytoplasm. In other areas, there was a proliferation of glands lined by columnar cells or squamous epithelium. Immunohistochemistry showed positivity for AE1/AE3, α-fetoprotein, SALL4, and glypican 3 (Figure 2). The findings led to the conclusion that the tumor was a germ cell yolk sac tumor (formerly known as an endodermal sinus tumor) with a mature teratoma component.

Chest and abdominal CT did not detect metastatic disease, so the patient was classified as stage III according to the Children Oncology Group (COG) staging criteria (Table 1).

**Table 1:** Staging (COG) of extragonadal germ cell tumors in children and adolescents. * Nodes measuring between 1 and 2 cm require follow-up for 4 to 6 weeks. If the node size remains unchanged (between 1 and 2 cm), a biopsy should be considered, or chemotherapy may be started. If the node increases in size, initiate chemotherapy. Any tumor involving the abdominal cavity or retroperitoneum should undergo analysis via peritoneal fluid cytology or peritoneal lavage, ensuring no malignant cells are detected. COG: Children Oncology Group.

Stage	Extent of Disease
I	Tumor completely resected at any site, including coccygectomy for sacrococcygeal sites. Tumor resected with negative margins and intact capsule. Any tumor involving the abdominal cavity. Negative peritoneal lavage by cytology for tumor cells. Lymph nodes ≤1 cm by imaging of abdomen, pelvis, or thorax*.
II	Completely resected tumor, but with preoperative or intraoperative biopsy, microscopic residual, or pathologic evidence of tumor capsule rupture. Microscopic residual. Imaging shows negative lymph nodes in abdomen, pelvis, and thorax. Peritoneal fluid cytology negative for tumor cells. Tumor markers positive or negative.
III	Biopsied or resected tumor with gross residual. Lymph nodes positive for malignant tumor cells, including immature teratoma. Lymph nodes positive with tumor resection. Lymph nodes ≥ 2 cm, or lymph node > 1 cm and < 2 cm by imaging (CT) that did not resolve on images taken at 4 to 6 weeks postoperatively.
IV	Distant metastases including liver, bone, lung, brain.

**Figure 3:**
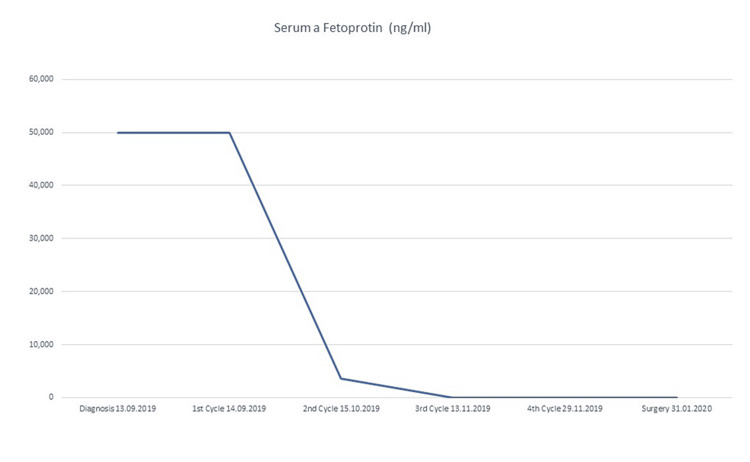
Serum alpha-fetoprotein levels during each cycle of pBEP treatment. pBEP: Bleomycin, Etoposide, Cisplatin.

**Figure 4:**
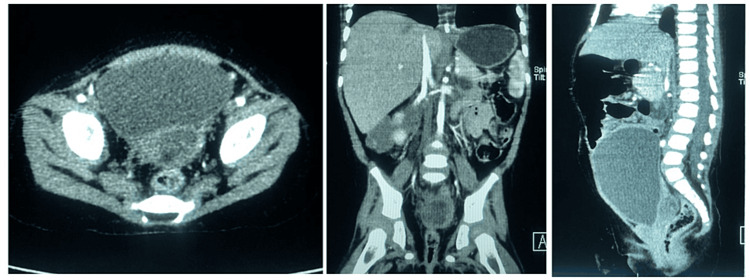
CT scan after chemotherapy. Abdominopelvic CT after four cycles of chemotherapy showing a decreased size of the mass. It appears heterogeneous with an irregular hypodense center and is located in the rectovesical septum, measuring 18x27 mm in the axial plane. There is no evidence of adenomegaly in the common iliac chains.

Neoadjuvant chemotherapy was initiated using a COG-based treatment strategy and protocol for GCTs in pediatric patients. The regimen, pBEP (Bleomycin 15 IU/m2 on day 1, Etoposide 100 mg/m2 on days 1 to 5, and Cisplatin 100 mg/m2 on day 1), was administered over four cycles with 21-day intervals. Imaging control and tumor marker assessments were conducted after each chemotherapy cycle. Figure 3 shows the trend of serum α-fetoprotein levels after each pBEP cycle as an assessment of response to chemotherapy. The pelvic CT was performed after four cycles of neoadjuvant chemotherapy, evidenced heterogeneous mass with irregular hypodense center and epicenter in the rectus vesical septum with dimensions in the axial plane of 18 x 27 mm ( approximately 90% reduction) without evidence of adenomegaly in common, internal, external iliac chains or the inguinal regions (Figure 4).

After an adequate response to chemotherapy, the patient was taken to surgery, where a 2 x 3 cm tumor was found on the anterior wall of the uterus extending to the uterine cervix with no evidence of retroperitoneal lymphadenopathy. Due to the intraoperative findings, the diagnostic was discussed with the parents, and it was agreed to perform a total hysterectomy. The histopathological report showed chronic multifocal xanthogranulomatous inflammation without evidence of viable tumor cells, which indicated a complete histopathologic response to chemotherapy. Currently, the patient is free of disease and is in periodic follow-up with negative tumor markers, with no evidence of new alterations in the images. 

## Discussion

GCTs are a heterogeneous group of benign or malignant neoplasms derived from primordial germ cells. They occur in various anatomical locations, predominantly in the gonads, but can also be found in extragonadal sites, mainly along the midline. These tumors can affect individuals of all ages, including neonates, infants, adolescents, and young adults, across both genders. In the pediatric age group, GCTs are rare; the incidence rate is 2.4 cases per million children, and they represent about 2% to 5% of all cancers diagnosed in children under 15 years. However, the incidence rate rises to 15% of all malignancies in individuals aged 15 to 19 years. The frequency of GCTs increases with age, peaking at 15% of all cancers between the ages of 15 and 19 years [1]. Moreover, the distribution of GCTs in children follows a bimodal pattern, with an initial peak from infancy until four years and a second peak in early puberty. They exhibit different molecular and clinical characteristics in each age group [5]. Histologically, it is a group of neoplasms that includes mature and immature teratomas, yolk sac tumors, embryonic carcinoma, choriocarcinoma, seminoma, and dysgerminoma [6]. Yolk sac tumor is a malignant neoplasm that resembles extra-embryonic structures, including the yolk sac, allantois, and extra-embryonic mesenchyme. It is the most common pure malignant GCT in children under two years and the only one to occur in the sacrococcygeal region in infants [7]. Extragonadal yolk sac tumors are infrequent in adolescents (only 0.6% of GCTs are composed of yolk sac tumors). Usually, it presents as a mixed component [8]. In our case, the presence of a yolk sac tumor was confirmed by elevated α-fetoprotein and immunohistochemistry. Additionally, this one showed a mature teratoma component located in the anterior wall of the uterus. The occurrence of this type of tumor is very infrequent; two systematic reviews reported 30 cases, a median age of 52 years, with a range of 24-87 years, and most of the patients were postmenopausal women [9,10]. Yin M et al. [4] reported a single case in children involving a 2-year-old girl. This report presents the second published case of a yolk sac tumor in the uterine corpus in a younger female infant. The child exhibited signs and symptoms similar to those reported in adult females, including vaginal bleeding and the presence of a pelvic mass, as detected by physical examination and imaging studies (ultrasound and CT or MRI of the abdominopelvic region). Given that transvaginal bleeding is a principal manifestation and may indicate various non-malignant pathologies such as anatomical malformations, infection, trauma, foreign body, sexual abuse, endocrinological disorders, hematological diseases, and pelvic mass, a thorough evaluation is necessary. Additionally, the presence of a pelvic or abdominal mass in prepubertal females necessitates an extensive assessment by healthcare professionals to exclude both benign diseases and malignant tumors. This includes considering rhabdomyosarcomas of the genitourinary tract and germ cell tumors of the uterus and vagina in the differential diagnosis.

Some MGCT subtypes secrete protein that can be valuable as a marker for the detection of the presence of a tumor; the elevation of these markers suggests a possible tumor histology. Particularly, the yolk sac tumor secretes a glycoprotein (α-fetoprotein) that may be detectable in serum at diagnosis but also serves to monitor the responses to therapy and post-treatment follow-up [6,11]. Figure 3 illustrates the α-fetoprotein curve starting at diagnosis and its decline after each cycle of chemotherapy until achieving normal pre-surgical values for our patient. The decline of serum levels until reaching negative values is the framework. 

The decrease in serum levels to normal levels is a good sign that neoadjuvant chemotherapy is working and an indication to perform a local primary tumor resection [3,12,13]. Generally, the management of MGCTs involves surgical treatment for cases that are resectable at diagnosis, followed by adjuvant chemotherapy. In the pediatric age group, the procedure often includes tumor resection, with an emphasis on fertility preservation, especially in cases of gonadal tumors. Additionally, as part of the staging process, it is recommended to collect peritoneal lavage samples for cytological study, palpate the peritoneal surface and omentum, examine the retroperitoneal lymph nodes, and biopsy any abnormal lesions observed during the procedure [14]. This type of tumor often presents challenges in removal at the time of diagnosis. Therefore, the initial surgical procedure is typically limited to performing a biopsy, followed by the administration of supplementary neoadjuvant chemotherapy to reduce the size of the tumor. This is then followed by a second-look surgery and possibly the administration of adjuvant chemotherapy. As previously reported in cases of unresectable uterine yolk sac tumors involving the uterine wall with an emphasis on fertility preservation [3,6,12], our patient had an unresectable tumor. It was initially biopsied through vaginoscopy, followed by four cycles of pBEP chemotherapy. The patient responded well to the treatment; however, uterine conservation was not possible, leading to a hysterectomy with preservation of the ovaries. After 46 months of clinical follow-up, including abdominopelvic ultrasound and tumor markers, the patient is free of disease, suggesting a favorable outcome.

## Conclusions

In summary, extragonadal yolk sac tumors located in the uterus are extremely rare in prepubertal girls and even more so in infants under two years old. As a result, it can be challenging to suspect and diagnose them. However, these tumors should be considered in the differential diagnosis of a pelvic mass with transvaginal bleeding and elevated serum alpha-fetoprotein levels for age. The treatment of these tumors is based on the guidelines for gonadal GCTs in girls. It includes conservative surgery with fertility preservation and chemotherapy. However, in cases where the tumors are unresectable at diagnosis, an initial biopsy, neoadjuvant chemotherapy, and surgery may be used. The use of pBEP chemotherapy has been shown to be effective for uterine yolk sac tumors.
